# Voxel Volumes and Biomass: Estimating Vegetation Volume and Litter Accumulation of Exotic Annual Grasses Using Automated Ultra‐High‐Resolution SfM and Advanced Classification Techniques

**DOI:** 10.1002/ece3.70883

**Published:** 2025-01-23

**Authors:** Josh Enterkine, Ahmad Hojatimalekshah, Monica Vermillion, Thomas Van Der Weide, Sergio A. Arispe, William J. Price, April Hulet, Nancy F. Glenn

**Affiliations:** ^1^ Boise State University Department of Geosciences Boise Idaho USA; ^2^ USDA US Forest Service, Forest Health Protection Region 4 Boise Idaho USA; ^3^ Oregon State University Extension Service‐Malheur County Oregon State University Ontario Oregon USA; ^4^ Oregon State University Extension Service‐Baker & Union Counties Oregon State University Baker City Oregon USA; ^5^ Plant & Wildlife Sciences Brigham Young University Provo Utah USA

**Keywords:** biomass, fine fuels, medusahead, rangeland, SfM

## Abstract

In much of the northern Great Basin of the western United States, rangelands, and semi‐arid ecosystems invaded by exotic annual grasses such as cheatgrass ( *Bromus tectorum* ) and medusahead ( *Taeniatherum caput‐medusae* ) are experiencing an increasingly short fire cycle, which is compounding and persistent. Improving and expanding ground‐based field methods for measuring the above‐ground biomass (AGB) may enable more sample collections across a landscape and over succession regimes and better harmonize with other remote sensing techniques. Developments and increased adoption of unoccupied aerial systems (UAS) and instrumentation for vegetation monitoring enable greater understanding of vegetation in many ecosystems. Research to understand the relationship of traditional field measurements with remotely sensed data in rangeland environments is growing rapidly, and there is increasing interest in the use of aerial platforms to quantify AGB and fine‐fuel load at pasture and landscape scales. Our study uses relatively inexpensive handheld photography with custom quadrat sampling frames to collect and automatically reconstruct 3D models of the vegetation within 0.2 m^2^ quadrats (*n* = 288). Next, we examine the relationship between volumetric estimates of vegetation with biomass. We found that volumes calculated with 0.5 cm voxel sizes (0.125 cm^3^) most closely represented the range of biomass weights. We further develop methods to classify ground points, finding a 2% reduction in predictive ability compared with validation ground surface reconstructions. This finding is significant given that our study site is characterized by a dense litter layer covering the ground surface, making reconstruction challenging. Overall, our best reconstruction workflow had an R^2^ of 0.42, further emphasizing the importance of high‐resolution imagery and reconstruction techniques. Ultimately, we conclude that more work is needed of increasing extents (such as from UAS) to better understand and constrain uncertainties in volumetric estimations of biomass in ecosystems with high amounts of invasive annual grasses and fine‐fuel litter.

## Graphical abstract

We use handheld photogrammetry to reconstruct vegetation and litter volume of semi‐arid rangeland vegetation, including invasive annual grasses and fine‐fuel litter accumulation. We developed a streamlined method to capture, process, and classify volumetric reconstructions, evaluated them as a nondestructive biomass measurement method, and investigate scalability of similar techniques to UAS collections.

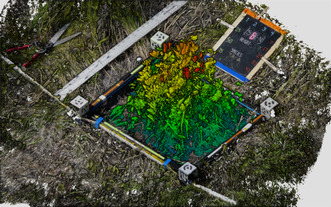

## 1 Introduction

Semi‐arid ecosystems act as carbon sinks and are thought to play a major role in global interannual carbon variations (Ahlström et al. 2015). In addition to soil carbon sequestration, semi‐arid ecosystems at regional scales provide important ecosystem services such as wildlife habitat and mesic refugia. Semi‐arid ecosystems, such as in the western U.S., are water‐limited, and pressures from climate change and population growth make these lands vulnerable to degradation. Specifically, the sagebrush‐steppe ecosystem is currently under threat from the invasion of exotic annual grasses, such as cheatgrass ( *Bromus tectorum* L.) and medusahead ( *Taeniatherum caput‐medusae* (L.) Nevski), which are decreasing biodiversity (Knapp 1996), altering the fire cycle (Bradley et al. 2018), and reducing carbon storage (Bradley et al. 2006).

Quantifying vegetation biomass is critical for assessing ecosystem structure, tracking vegetation growth, and quantifying carbon storage (Houghton, Hall, and Goetz 2009). Above‐ground biomass (AGB) in semi‐arid ecosystems helps scientists and land managers better understand the contribution of these ecosystems to the global carbon flux, and the impact of ecosystem shifts toward desertification (Chambers et al. 2014). In particular, AGB is defined as the dried weight of vegetation above the ground including both alive and dead components and is difficult to accurately measure in semi‐arid ecosystems because of the heterogeneity and fine‐scale structure of vegetation (Fern et al. 2018; Wijesingha et al. 2019). This is especially so for invasive annual grasses such as medusahead and cheatgrass, where previous growth can become a mat of dry litter, decreasing native vegetation growth and further promoting the invasive annual grass species (Evans and Young 1970). As a result, an increase in the continuity of fine‐fuel loads occurs. This build‐up of fine fuels, coupled with senescence early in the growing season, promotes ignition and increased fire spread. This feedback loop, “invasive grass‐fire cycle,” further results in the degradation of semi‐arid ecosystems (Fernández‐Guisuraga et al. 2022).

Management approaches to the invasive grass‐fire cycle are varied across techniques and spatial and temporal scales. Such approaches may include re‐seeding, grazing, and fuel breaks at the plot, pasture, or regional scales (100s–1000s km^2^, e.g. Price et al. 2023). Where the sites may be relatively small in extent (e.g., grazing exclosure scales, or m to km), there is a benefit of reducing the need to destructively harvest samples to observe changes while reducing the impact on the vegetation. High‐resolution monitoring data with low uncertainty are needed to provide baseline information at both scales, enabling more accurate extrapolation of measurements. This is particularly valuable for observing changes over time in response to treatments and climate.

Current methods for collecting AGB can be described as a combination of site‐specific and extrapolated measurements. An example of site‐specific measurements is the destructive harvesting of biomass over small areas (e.g., 0.25–1 m^2^), which are then extrapolated to larger scales using other field observations or remote sensing datasets (Li et al. 2017). Collecting biomass datasets involves hand clipping, sorting, drying, and weighing components to obtain a biomass measurement; occasionally, quadrat point frame measure data (pin‐drop counts) are also used to relate to biomass (Clark et al. 2008). Often, these plots are small (e.g., 0.25–1 m^2^), although the biomass in semi‐arid ecosystems can vary significantly across spatial scales, even within 1 m or less. There are also limitations in the size–biomass allometry assumption, especially related to climate (Rudgers et al. 2019) and environmental or phenological conditions (Muldavin et al. 2008).

The act of removing vegetation and litter itself alters the landscape and impacts future studies of those plots and is subject to some influences of collection processes. Advances in remote sensing systems and methods using lidar and photogrammetry/Structure from Motion (SfM) from spaceborne, airborne, unoccupied aerial systems (UAS), and terrestrial systems have led to advances in quantifying biomass in dryland ecosystems (Anderson et al. 2018; Cunliffe, Brazier, and Anderson 2016; Fernández‐Guisuraga et al. 2024). Fine (spatial/structural) resolution remote sensing has the potential to help solve the above rangeland quantification challenges.

While both SfM and lidar (from any platform) typically produce point clouds, SfM is a photogrammetry technique that uses passive optical 2D images (often uncalibrated) to detect common points across multiple images and may not penetrate the canopy (Salamí, Barrado, and Pastor 2014). Lidar uses active sensing, emitting laser pulses and measuring the time it takes for them to return, thereby providing precise 3D coordinates for each point even through dense vegetation. Lidar can be collected in full‐waveform or with multiple discrete returns, allowing it to better characterize the understory and ground surface (Wallace et al. 2017). Lidar also captures intensity, which refers to the amount of light reflected from each point. SfM point clouds similarly capture a measure of intensity information in the form of red, green, and blue values from the optical imagery.

Vegetation parameters such as volume can be derived from point clouds to develop allometric relationships between vegetation and destructively harvested biomass. SfM offers a low‐cost, time‐efficient, and in some cases more accurate method compared to terrestrial laser scanning (TLS) for estimating vegetation structure in dryland ecosystems (Cooper et al. 2017; Olsoy et al. 2018; Wallace et al. 2017, Taugourdeau et al. 2022).

Extremely close‐range SfM, here defined by the use of cameras held by an operator, has shown success comparable to TLS in deriving parameters such as height and volume from the reconstruction of individual trees (Miller, Morgenroth, and Gomez 2015). In a grassland study, extremely close‐range SfM was shown to outperform TLS in the measurements of above‐ground biomass (AGB) of grass (*R* ^2^ = 0.54 and *R* ^2^ = 0.46, respectively), in part because SfM can capture finer details compared to TLS (Cooper et al. 2017). The use of close‐range SfM from videography to model grass biomass in a forest understory showed a strong correlation (*R* ^2^ = 0.76 of SfM volume to grass biomass) (Cova et al. 2023). These previous studies highlight the potential of SfM to provide accurate information for allometric equations used to extrapolate the biomass of grass at plot scales.

Field work for capturing extremely close‐range SfM imagery requires less training for field crews compared to TLS and UAS data collection and eliminates the need for specialized and costly equipment. Depending upon the experimental setup, SfM can also avoid some of the challenges of occlusion in grass and shrublands that TLS encounters (e.g., Anderson et al. 2017). Furthermore, developing allometric relationships using close‐range SfM of similar scales of measurement to UAS data (e.g., cm‐level measurements) can be a complementary precursor for extrapolating to areas at scales covered by UAS (e.g., 1000s of m^2^ to 10s of km^2^; Gillan et al. 2019). However, vegetation and soil classification in such a fine resolution dataset remain challenging. Spectral reflectance in the visible spectrum and near‐infrared may be able to better separate vegetation and dry biomass litter from one another, or from the mineral soil or ground surface. Although differences in the soil composition can cause different types of confusion with vegetation and litter, possibly reducing transferability of methods between study sites (Huang et al. 2007), in areas where the ground surface can be largely obscured, may benefit from other approaches such as structural analyses from either SfM or active remote sensing technologies (Calders et al. 2015).

A common technique for the estimation of AGB relies on Digital Surface Models (DSMs, the elevations of retrieved data, including vegetation) and Digital Elevation Models (DEMs, or the ground surface) to compute the retrieved Canopy Height Model (CHM) which is the difference between them. (Ota et al. 2015; Zhang et al. 2018; Xu et al. 2020). Airborne and UAS lidar and UAS SfM‐based derivations of CHM generally classify ground and vegetation by setting a height threshold (Cunliffe, Brazier, and Anderson 2016; Viljanen et al. 2018; Näsi et al. 2018; Navarro et al. 2020). Fernández‐Guisuraga et al. (2024) used a progressive triangulated irregular network algorithm on the lowest UAS lidar points within 20 cm rasters to create a DEM which was then used to normalize the DSM to create a CHM.

While CHMs are considered 2.5‐dimensional data (i.e., a raster where the cells represent a height), voxels are a three‐dimensional representation of volume using cubes (“volumetric pixels”), and therefore a vertical column may have a mix of empty and filled spaces. Convex hulls are another computational method to calculate volumes, by calculating the smallest convex boundary that fully encloses a set of points or features, much like wrapping a cloth around a shape.

Schulze‐Brüninghoff, Wachendorf, and Astor (2021) suggested a method combining lidar‐derived metrics (sum of voxels, canopy height model, and canopy surface structure) and vegetation spectral properties to estimate fresh and dry biomass in a machine learning approach. Fernández‐Guisuraga et al. (2024) found that using UAS‐lidar to characterize the vertical plant structure below the top of the canopy and finely resolved ground surfaces was critical to estimating biomass fractions in the study area of this paper. Together, uncertainty of biomass estimations is limited to the uncertainty in CHM or vegetation heights, data resolution, and the type of land treatments or cover (such as thick layers of vegetative litter). In this regard, the discrimination between vegetation and bare ground structural properties (smoothness of the surface, randomness of vegetation foliage, and curvature) could improve AGB estimation from high‐resolution ground/close‐range camera and UAS SfM‐derived point clouds.

The objective of this work is to explore the potential for close‐range SfM to quantify fine‐fuel AGB, with the goal to evaluate replacing destructively harvested sampling with the methodology, or to utilize the close‐range SfM volumetric estimations to inform potential UAS data collection. To accomplish this, we first developed a method to acquire and process field data from photos to point clouds. Second, we explored how to separate the ground surface from the thick litter layer found in the study area. Third, we examined the relationship between the measurement method (CHM, convex hulls, and voxel volumes) and resolution of volumetric calculation and the biomass of the study area's vegetation. Lastly, we discuss how a SfM quadrat sample frame might be used, and how this method may inform above‐ground biomass or vegetation volume estimates with UAS data.

## 2 Methods

### 2.1 Study Area

**FIGURE 1:**
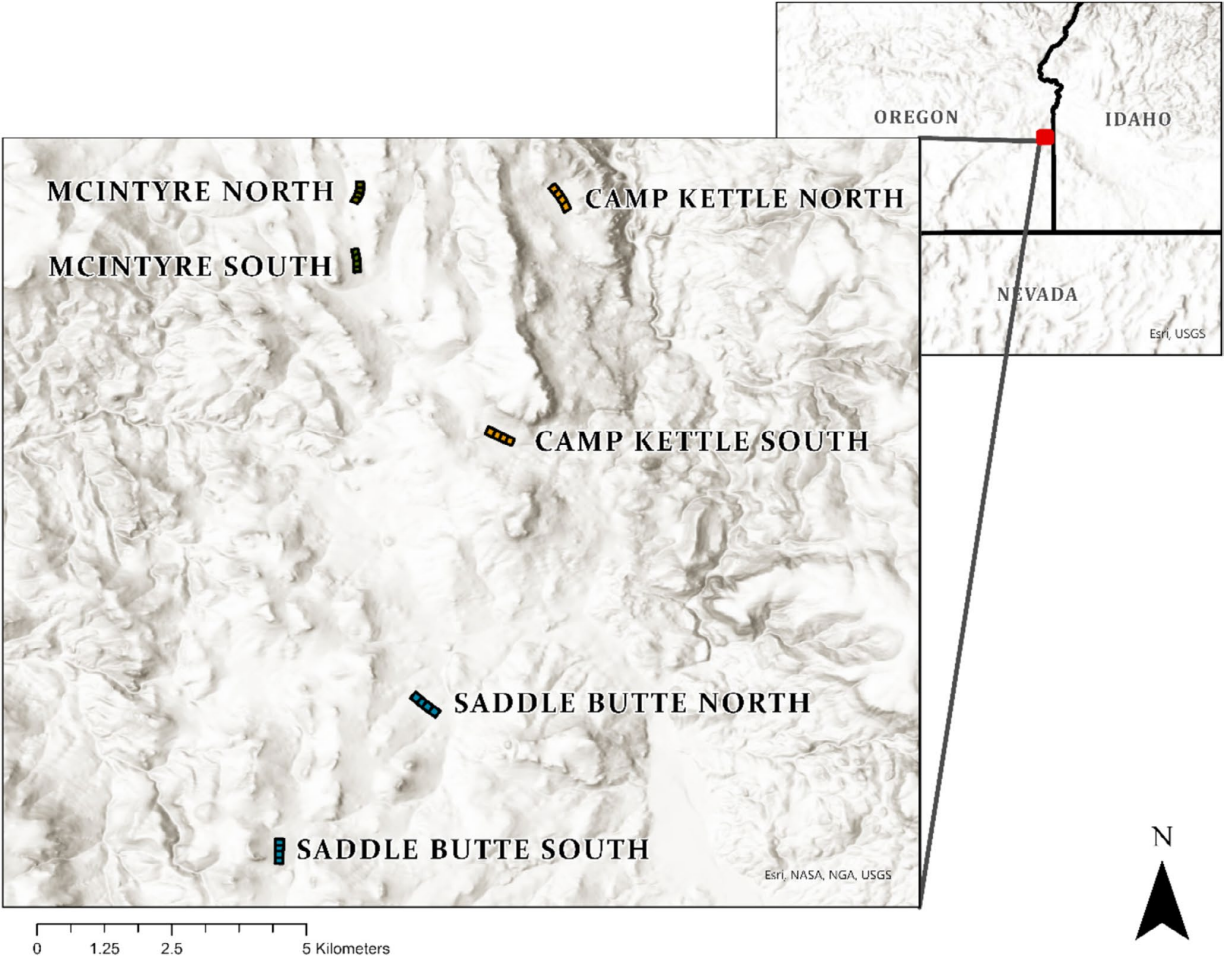
Study area in southeast Oregon, USA. At each pasture, two sites were established for a total of six treatment areas. At each of the six treatment areas, four paddocks (150 × 150 m) were established; within each paddock, three transects are established annually. Each transect is sampled in three places with destructive harvesting and Structure‐from‐Motion volumetric estimates.

Our study area is the Three Fingers allotment located in southeastern Oregon (Figure 1), approximately 80 km west of Boise, Idaho. The Vale District U.S. Bureau of Land Management (BLM) manages the allotment, which has a history of disturbance from grazing, recreation, and fire. The area has experienced significant departure from native vegetation communities, with few remaining native shrubs and an extensive cover of cheatgrass and medusahead, and other annual grasses (> 80% in all experimental exclosures).

This study was part of a larger study focused on evaluating the effect of dormant season grazing on fine fuels (Arispe et al. 2022). As part of the project, data were collected across 2 years and on three different pastures on the Three Fingers allotment—approximately 55,000 ha. The three pastures within the project included McIntyre (MCI; 3100 ha), South Camp Kettle (SCK; 2500 ha), and Saddle Butte (SB; 3800 ha). Within each pasture, a northern and southern exclosure was randomly placed, for a total of six treatment areas. Each exclosure contained four paddocks 150 × 150 m in size, for a total of 24 paddocks.

### 2.2 Field Data and Collection Protocol

Data were collected in June 2020 and June 2021, as a supplement to existing protocol (Arispe et al. 2022) for collecting biomass data in each of the 24 experimental paddocks. For each of the 24 paddocks, three transects are established from a randomized central location. Along each transect, three 0.4 m by 0.5 m areas were photographed for Structure‐from‐Motion reconstruction and then destructively harvested. Vegetation was removed to as near to mineral soil as possible and separated into annual grasses, perennial grasses, forbs, and litter prior to drying. Plots that contained shrubs were excluded, since shrubs are not destructively harvested for the larger project. Samples were oven‐dried for 48 h at 60°C in a commercial drying hood and weighed (35.3–4127.1 g, μ = 893.8 g for those used in this study). Due to field time constraints, approximately ⅓ of all plots were photographed again immediately after harvesting the vegetation but before removing the quadrat sample frames in order to capture the mineral soil microtopography to use as validation reference surfaces of our ground classification methods. Large nails were used to ensure the frame did not move during the AGB collection process. After automated reconstruction and quality control, approximately ⅙ (*n* = 35) remained of the post‐AGB collection plots.

### 2.3 Structure‐From‐Motion Protocol and Reconstruction

**FIGURE 2:**
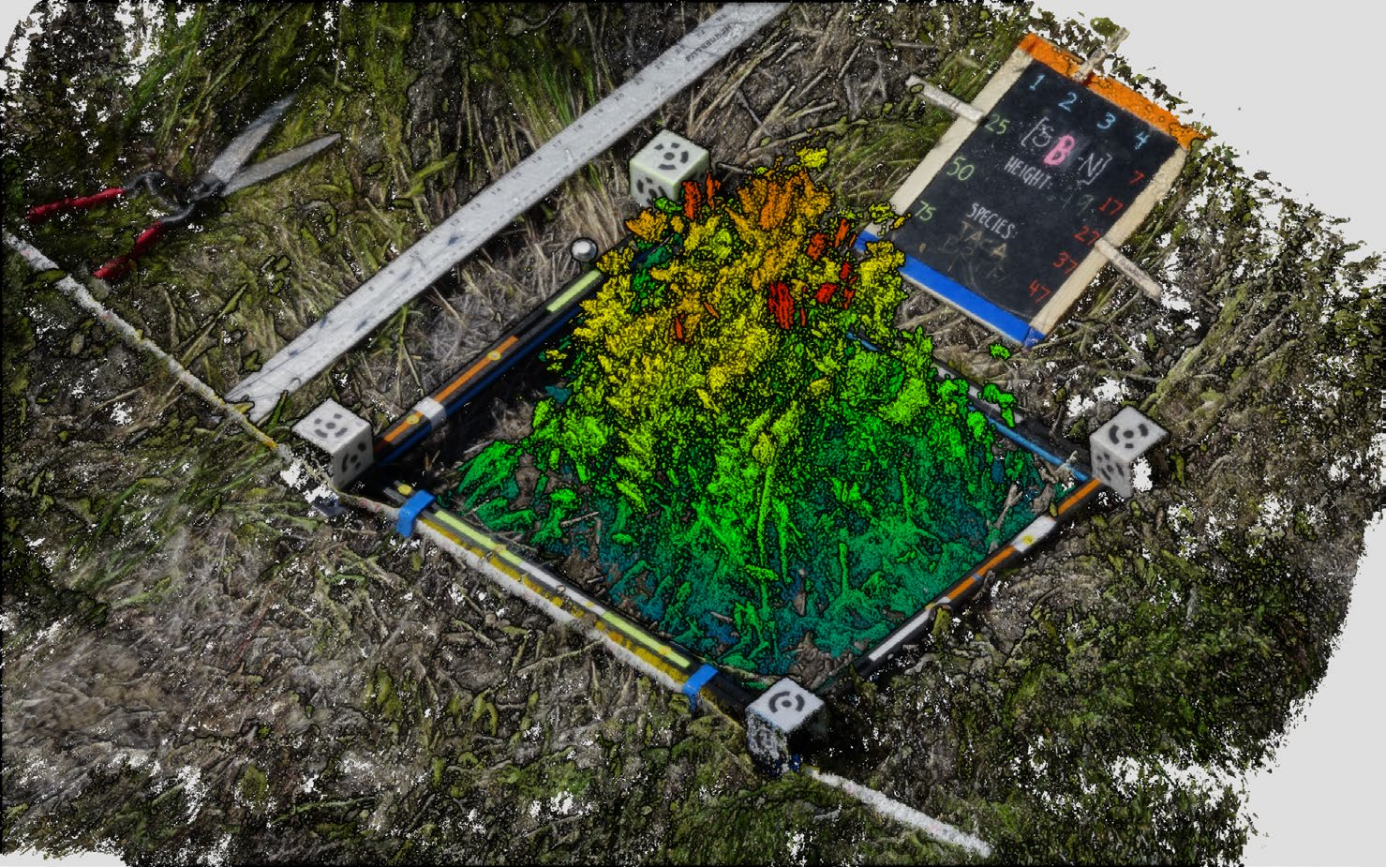
Point cloud reconstruction of a typical 0.4 × 0.5 m plot before destructive harvesting, including the SfM quadrat sample frame (black with color inlays) with coded targets (white cubes, 125 cm^3^). At regular intervals, the quadrat sample frame was placed on the mineral soil surface taking care to avoid crushing or flattening vegetation or compressing litter. Vegetation (primarily Taeniatherum caput‐medusae) is colored by height in this visualization.

Two custom quadrat sample frames were constructed from extruded aluminum, containing custom 3D‐printed 0.05 m cubes with coded targets (Figure 2). These targets are automatically detected in Agisoft Metashape (version 1.6). We used a Sony a6000 with the stock kit lens (16–50 mm F3.5–5.6, set to a fixed focal distance of 24 mm at 35 mm equivalent) to collect approximately 75 photographs per quadrat sample frame. These were taken approximately 1 m above the plot in a 5 × 5 sampling pattern repeated three times: Once at nadir, once at approximately 20° tilt away from the photographer, and again tilted toward the photographer. Ground sample distances at 1 m is 0.37 × 0.16 mm (width × height). Several additional images were taken at each plot from a greater height and at several corner‐on angles. Care was taken to stand so as to avoid casting a shadow on the plot, as photos were collected in the morning and through to solar noon. One of each of the six sites was collected per day. In total, approximately 288 (after reconstruction) plots were used in our analysis across the treatment areas in both 2020 and 2021.

Our processing workflow in Agisoft Metashape (version 1.6) was automated using Python; after estimating camera parameters and removing images with an estimated quality of < 0.7, each plot was processed at high‐quality image alignment and high‐quality dense cloud reconstruction with iterative sparse point filtering; additional processing parameters are described in the processing script as part of our Open Data Statement (see Enterkine, Hojatimalekshah, and Glenn 2024). Resulting point clouds had typical densities of 0.5 to 4 million points per square meter filtered to a minimum of 0.001 m spacing between points. After processing and quality assessment, there were 114 plots for 2020 and 174 plots for 2021.

### 2.4 Point Cloud Classification and Volumetric Processing

#### 2.4.1 Preprocessing Point Clouds and Ground Classification

**FIGURE 3:**
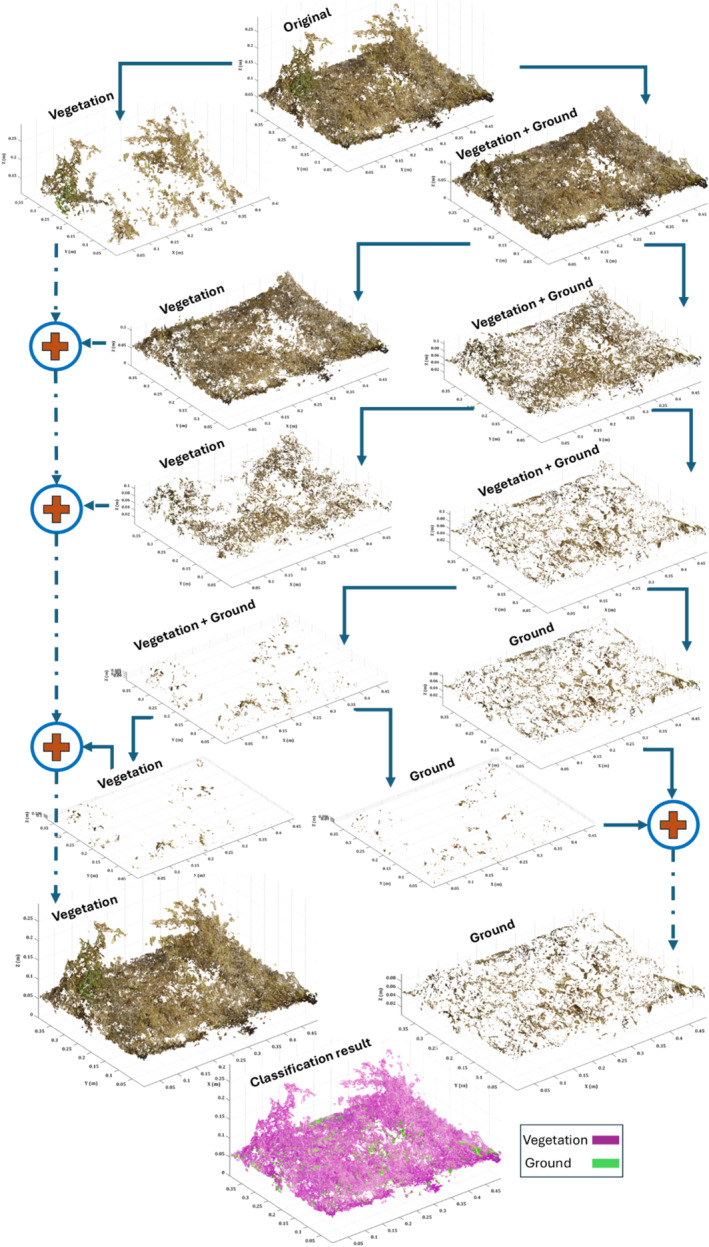
Schematic workflow for point cloud classification of an example plot. The iterative steps are shown with blue arrows, and additive grouping of similarly classified points is shown with red “plus” signs.

Following reconstruction, point clouds were imported into Matlab (version R2021a) for classification into ground and vegetation classes and volumetric measurements (Figure 3). After clipping the point clouds inside the quadrat sampling frames, we applied the point cloud denoising algorithm presented by Rusu et al. (2008) to remove the outliers from the point cloud. To classify the point cloud into ground and vegetation classes, we applied the simple morphological filter (SMRF) algorithm in Matlab.

The simple morphological filter (SMRF) method (Pingel, Clarke, and McBride 2013) classifies the point clouds into vegetation and ground using the elevation and slope threshold. The algorithm tiles the data by a grid resolution, finds the minimum elevation points within each grid, and fits a surface to the minimum points. The points that are excluded from the elevation difference between the minimum surface and morphological opened minimum surface by linearly increased window sizes (from 1 to user‐defined maximum window radius) in an iterative procedure are considered as vegetation and the points that satisfy the elevation threshold are classified as ground (more detail can be found in the study of Pingel, Clarke, and McBride 2013).

We used an elevation threshold of 11 cm, slope threshold of 0.1 (as the plots were mostly flat), elevation scale of 0.9 (to detect medium‐size objects), and max window radius of 10. While this is effective, the ground classified points still contain some vegetation biomass. To separate the remaining vegetation points from the ground class, we applied two Gaussian mixture classification algorithms on the curvature of the point clouds using 50 and 200 neighbors (200 neighbors are applied on the ground class from 50 neighbors’ output). In this method, we assume that the ground surface is smoother than the vegetation surface, and thus the curvature on the surface is different for those classes. The algorithm extracts curvatures and fits a Gaussian distribution and assumes vegetation and ground curvatures follow two mixed Gaussian distributions. After retrieving the vegetation and ground class, we merged the vegetation class with the previous vegetation class from the SMRF results. The classification criterion relied on the enhancement of vegetation volume. If an improvement of more than 5% was not observed, the algorithm terminated. Through a comprehensive point cloud classification, we determined that employing two Gaussian mixture clusters based on point cloud elevation yielded optimal improvements in vegetation volume and processing time for our classification algorithm.

#### 2.4.2 Volumetric Processing: Canopy Height Models

We applied the method described by Cunliffe et al. (2022) to compute the canopy height models (CHMs). To estimate the mean canopy height within a specified plot, we first calculated the height above ground for each point in the point cloud by subtracting the corresponding terrain elevation. This was done by associating each point with the underlying terrain model at a 0.01 m resolution. Next, we created a grid matching this resolution and assigned the maximum canopy height to each cell based on the points within it. For cells without points, we applied inverse distance weighting to interpolate canopy heights from nearby cells within a 7 × 7 neighborhood. If no neighboring cells contained data, the cell remained empty. Finally, the plot‐level mean canopy height was calculated by averaging the heights of all non‐empty cells in the grid. This method ensures that gaps in the data are addressed through interpolation, providing a reliable estimate of the average canopy height for the plot. We chose to use these values in our modeling as they are equivalent to volume: Multiplying by the horizontal area of the sample quadrat frame (0.2 m^2^) would yield a measure in m^3^.

#### 2.4.3 Volumetric Processing: Convex Hulls

Convex hulls represent volume by calculating the smallest convex boundary that fully encloses all the nonground points of each plot. To compute this, we used the pcdenoise function in Matlab (R2021a) to remove outliers from the point cloud data. We applied a threshold of 0.1, where the threshold corresponds to one standard deviation beyond the mean of the average distances between each point and its k‐nearest neighbors. If a point's average distance to its neighbors exceeded this threshold, it was marked as an outlier. By removing these outliers, we reduced noise in the data, ensuring that the convex hull will be computed from a cleaner, more accurate dataset. Next, we employed the boundary function in Matlab (R2021a), which generates a tight‐fitting boundary around the point cloud. By setting the shrink factor to 1, we ensured that the boundary was the convex hull itself, without additional shrinking. This approach provides a broader approximation of the vegetation volume, as it includes all the points within the convex boundary, potentially including empty spaces.

#### 2.4.4 Volumetric Processing: Voxel Volumes

Voxel volumes represent the presence or absence of nonground points within each cell. For this, we applied the same denoising technique used for convex hull volume computation. We divided the point cloud data into cubic bins (voxels), each with dimensions of sizes ranging from 2 to 100 mm on a side. By counting the number of voxels containing at least one nonground point, we were able to approximate the total nonground (i.e., vegetation + litter) voxels; multiplying the number of occupied voxels by the volume of each individual voxel yields a calculated volume. This method provides a fine‐grained, high‐resolution estimate of the vegetation volume based on the spatial distribution of points in the cloud.

### 2.5 Evaluating Volumetric Reconstruction Methods

Once the data were classified into ground and vegetation, we investigated several methods to compare the calculated volume with AGB. We examined using all points that were not classified as ground and using all nonground points that remained above the interpolated mesh surface. Additionally, we examined using combinations of the different classes of AGB (annual grasses, perennial grasses, forbs, and litter) to investigate if our reconstructions were biased toward a particular morphology. Further, we compared convex hull calculations and CHM calculations with voxel volumes and investigated the impact of voxel sizes on the observed relationships with AGB.

Convex hull analysis compared the relationship between biomass and vegetation volume using the convex hull volume of the classified nonground (i.e., biomass) points. Both linear and logarithmic models were explored, and we evaluated the regression fit by calculating the root‐mean‐square error (RMSE) and the coefficient of determination (*R* ^2^).

We assessed the scale at which volumetric voxel data are best reconstructed for sagebrush‐steppe vegetation (e.g., fine annual grasses) to inform future UAS imagery collection design parameters. Our analysis included voxelizing the point clouds at several resolutions ranging from 2 to 100 mm on a side (i.e., 0.8–1000 cm^3^ voxels), where the presence of a single SfM point counted as filling the voxel. These distributions of measured volumes of plots were compared with the distribution of biomass to determine which voxel size appropriately captured the structure of fine grasses and litter.

Next, we compared our method of classifying ground surfaces in environments with fine grasses and thick layers of litter from SfM. This was accomplished by comparing volumes of nonground voxels when removing ground points from our surface classification and those from the SfM‐reconstructed mineral soil surfaces collected after harvesting biomass.

We assessed the data based on per site (*n* = 6) and as combined sites per year, and by combining years. Our interest in assessing on a per‐site basis was to consider possible effects of changing photography conditions (one site photographed per day) or influences of the site location such as general vegetation type or subtle differences in environmental conditions. A similar logic was applied to the per‐year versus both years combined datasets. Additionally, we examined the relationships between volumes and vegetation type (e.g., perennial and annual grasses, forbs). Linear models and logarithmic models were both investigated for the relationships between voxel volume and biomass. Ultimately, in this model, litter was considered as part of the biomass volume.

## 3 Results

### 3.1 Canopy Height Model and Convex Hull Volumetric Reconstructions and Biomass Allometry

Table 1 lists the significant (> 0.30 *R* ^2^) findings from the CHM values and Convex Hull volumetric relationships (*R* ^2^ values of all comparisons are listed in Table S1). The *R* ^2^ value for the linear regression between CHM and biomass was 0.25, while the logarithmic regression yields an *R* ^2^ of 0.35. A statistically significant *R* ^2^ value was observed for a logarithmic relationship between he tvegetation convex hull volume above the reconstructed ground surface and total biomass (*R* ^2^ = 0.42). Generally, our vegetation voxel volume calculated from classified vegetation points (from Figure 3, workflow) performed better than using a fitted convex hull or the CHM‐based volumes (Table 2). Given these findings, we chose to focus primarily on the voxel volume results, as they provided a clearer and more consistent relationship with biomass.

**TABLE 1:** Retrieved *R* ^2^ and RMSE by applying a linear and logarithmic regression of CHM and Convex Hull volume estimates vs. biomass (dried weights; AG = annual grass, PG = perennial grass). Vegetation volumes of all points that are not classified as ground points, in contrast to only nonground voxels above the interpolated mesh ground surface, as classified by the workflow (Figure 3). The reported values are the significant ones according to the *f* ^2^ test for the sample size. The minimum threshold for power is 90%.

Comparison	Linear regression *R* ^2^ and RMSE (g)	Logarithmic regression *R* ^2^ and RMSE (g)
Canopy Height Model vs. vegetation AGB (AG + PG + Forb)	0.25, 620	0.35, 621
Vegetation Convex Hull volume vs. vegetation AGB (AG + PG + Forb)		0.41, 651
Vegetation Convex Hull volume above reconstructed ground surface vs. total AGB		0.42, 642

**TABLE 2:** Retrieved *R* ^2^ and RMSE by applying a linear and logarithmic regression of voxel volume estimates vs. biomass (dried weights; AG = annual grass, PG = perennial grass). Vegetation voxel volume is the voxelized volume of all points that are not classified as ground points, in contrast to only nonground voxels above the interpolated mesh ground surface, as classified by the workflow (Figure 3). The reported values are the significant ones according to the *f* ^2^ test for the sample size. The minimum threshold for power is 90%.

Comparison (5 mm voxel)	Linear regression *R* ^2^ and RMSE (g)	Logarithmic regression *R* ^2^ and RMSE (g)
Vegetation voxel volume vs. vegetation AGB (AG + PG + Forb)	0.35, 603	0.48, 621
Vegetation voxel volume vs. vegetation AGB + litter		0.32, 1674
Vegetation voxel volume above reconstructed ground surface vs. total AGB	0.30, 624	
Vegetation voxel volume vs. AG + PG AGB	0.33, 576	0.40, 599

### 3.2 Voxel Volume Reconstruction Scales and Biomass Allometry

**FIGURE 4:**
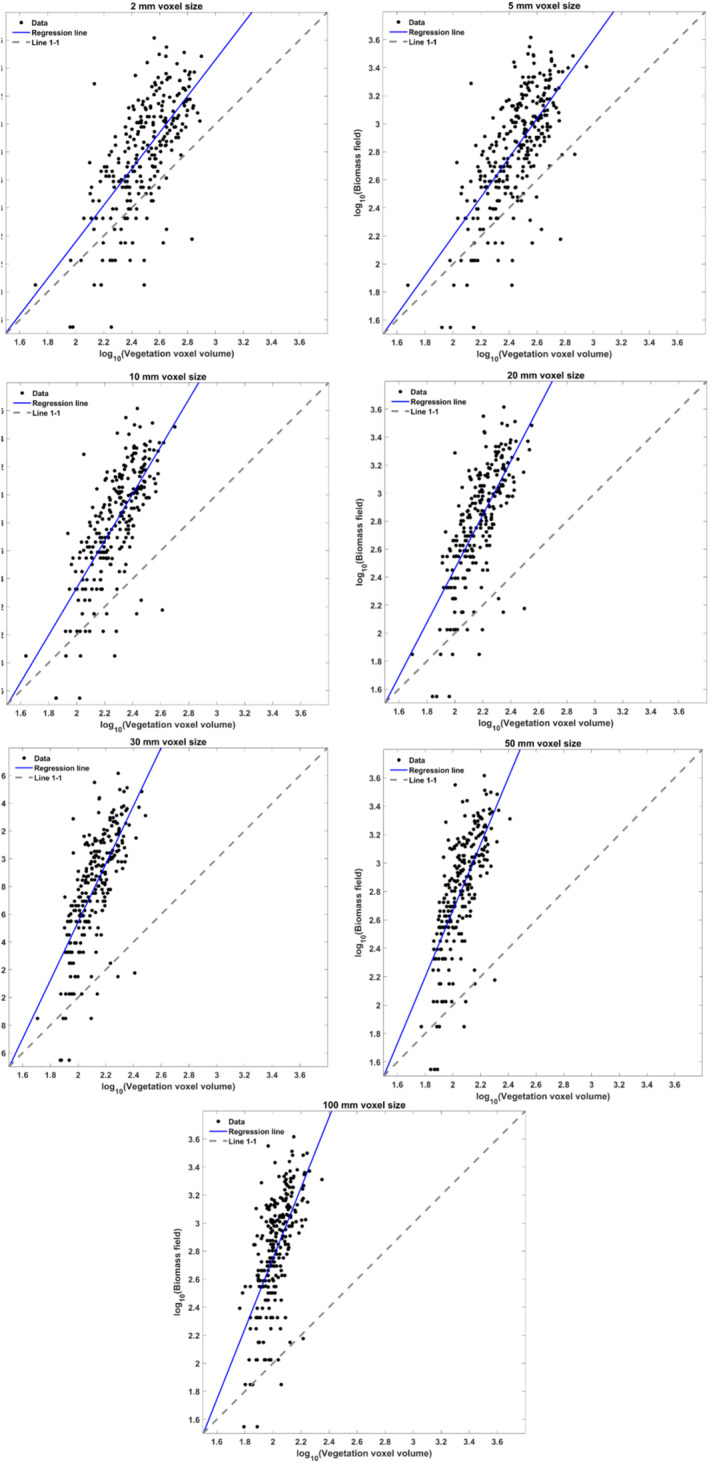
Scatter plots of the log of vegetation (nonground) voxel volume and log of biomass at various voxel sizes for 288 plots. The blue line represents the regression line, while the gray dashed line indicates a perfect correlation.

**FIGURE 5:**
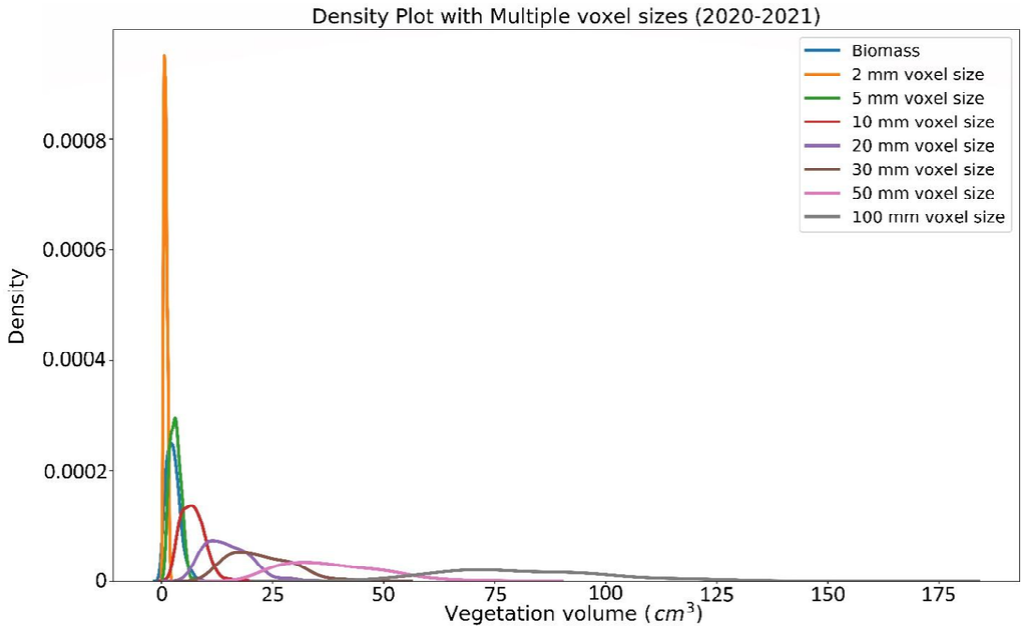
Biomass and vegetation volume distribution at different voxel sizes for 2020–2021 data. This figure illustrates the distribution of calculated voxel volumes per plot and the values of the biomass measurements. Smaller voxel sizes record a less varied distribution and scale of vegetation volumes for all samples, and larger voxel sizes result in the overall larger calculated vegetation volumes with a larger range of sizes. The mean and standard deviation of voxel volumes at 5 mm voxel sizes are the most similar to those of the AGB values.

Investigating the effects of volumetric resolution on data distributions was conducted by voxelizing the point clouds in intervals from 2 to 100 mm. Figure 4 illustrates the relationships between different voxel sizes (of nonground points) and the biomass. The non‐normal distributions of calculated volumes with the differing voxel sizes were all statistically different from one another (Figure 5), as determined by Wilcoxon tests.

### 3.3 Reconstructed Classified Ground Surface Validation Using Reference Surfaces

**FIGURE 6:**
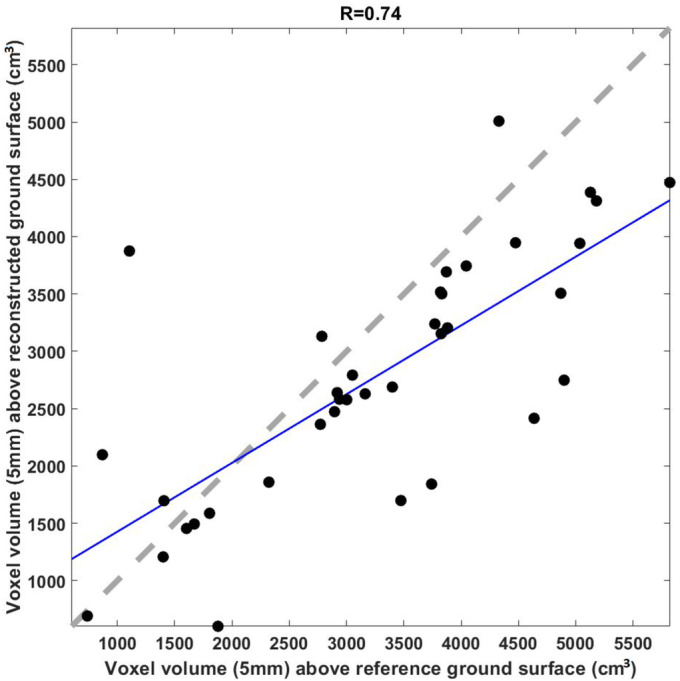
Calculated voxel volumes of nonground points (vegetation and litter) using reconstructed estimated ground surfaces with methods described in Section 2.4 compared with the reference ground surfaces reconstructed with SfM (*n* = 35) after destructively harvesting vegetation and litter. The blue line represents the Total Least Squares regression between the calculated voxel volumes, while the dashed gray line indicates a perfect correlation. Lin's Concordance Correlation Coefficient is 0.67 (Lawrence 1989; Lawrence 1992).

To evaluate errors in our vegetation volume calculations due to our ground classification method, we compared vegetation volume calculations at 5 mm voxel sizes using the plots where we collected SfM imagery after destructive harvesting (*n* = 35). Figure 6 illustrates the relationship between the volumes above the reconstructed surface and the reference surface. These bare mineral soil surfaces are considered the “true” reference ground surface. Nonground (vegetation + litter) volumes calculated using our reconstructed interpolated ground surface showed a decrease in *R* ^2^ from 0.44 to 0.42 (5 mm voxels). The mean absolute difference in volumes was 20% of the volume calculated using the reference ground surface.

### 3.4 Voxel Volume Relationships With Vegetation Types by Biomass

Table 2 depicts the models we tested that had a significant (> 0.30 *R* ^2^) relationship between voxel volume and dry weight biomass (g; Table S1 contains all values). We found the best correlation (*R* ^2^ = 0.48) with a logarithmic regression between 5 mm voxel (0.125 cm^3^) volumes of vegetation‐classified points with the above ground biomass for all sites across years (Table 2). We generally found that logarithmic models performed better than linear models when all plots were grouped together across years. No relationship between the voxel volume and forb biomass was found due to their relative scarcity by biomass in the study area.

A linear regression of plots (*n* = 36) from both years from South Camp Kettle (South site) attained an *R* ^2^ of 0.62 (RMSE of 281 g) between classified vegetation points at 5 mm voxel sizes compared to the biomass of only annual and perennial grasses (i.e., no litter or forb biomass included). However, generally, there was no pattern of linear or logarithmic modeling performing better for any of the comparisons when modeling by site or by year.

## 4 Discussion

The outcome of this research illuminates some considerations specific to the vegetation of the study area, chiefly the gracile structure of medusahead and the layer of litter that it deposits. Broadly, we found that the largest cause for uncertainty in our estimations resulted from the fine‐fuel litter layer obscuring the ground surface. However, with additional processing, we were able to attain some informative results.

### 4.1 Structure‐From‐Motion Quadrat Sample Frame and Reconstruction

Reconstructing point clouds from the field photography is a feasible sampling strategy for studies of this scale. However automation should be employed as much as possible, since reconstruction of point clouds can be labor‐ and processing‐intensive. The addition of coded targets at known, local coordinates greatly reduces the need for manual steps in the process. We observed that the reconstruction success of the point clouds was mostly dependent on the photo quality (e.g., not blurry), amount of overlap, and that the targets were not obstructed by even a small amount of vegetation.

Environmental conditions also played a role in the success of the point cloud reconstruction; occasionally, lighting conditions created too many shadows or too much variability during the photographing process. This caused more issues with the reconstruction of a sample quadrat frame where lighting changed more drastically during the image collection and caused reconstructions to fail. Slade et al. (2024) found that changes in sun elevation had little effect on reconstructed canopy heights, but wind did. Due to the relatively delicate structure of the vegetation of our study site, small gusts of wind can cause a plot to fail reconstruction quality thresholds or fail to process altogether likely due to too much variability in the positions of grass stems. One advantage of a camera operated by a human, as in our study, is the ability to pause data collection briefly to let clouds and wind gusts pass before resuming the collection of the imagery. Further refinements in SfM capturing techniques or similar visual representation techniques such as plenoptic learning (Neural Radiance Fields or “NeRFs” and Gaussian Splatting) use faster data collection methods such as short videos (Mildenhall et al. 2020; Kerbl et al. 2023), which may avoid some of the issues with gusts or lighting conditions.

### 4.2 Volumetric Estimation Methods and Scale

While the convex hull method may better handle the undersampled volumes internal to a mat of litter or dense vegetation, we found that although the results were similar to the voxel models, the convex hull method generally performed worse across the different classifications and comparison models when compared with the voxel volume method. Similarly, the CHM approach performed worse than the voxel volume with a linear relationship and similarly with a logarithmic relationship compared to linear and logarithmic relationships between voxel volumes and biomass.

Although not discussed in the Methods 2 or Results 3 Sections, we also explored calculations based on three divisions of Canopy Height Model (CHM) metrics and employed these, along with the convex hull volume and voxel volume, to train two random forest models for biomass estimation. The results indicated *R* ^2^ values of 0.36 (RMSE = 1058 g) and 0.45 (RMSE = 984 g) on 30% of the test data for CHM and convex hull volume, and CHM and voxel volume as independent variables, respectively. However, one objective of this study was elucidating the presence of linear or logarithmic relationships between vegetation volume and biomass to be more broadly applicable to future studies.

Because the distribution of volumetric calculations among plots were statistically significant for each voxel size based on the Wilcoxon tests, we ascertain that the volumetric calculations are highly dependent on the resolution of the final point cloud, which is constrained by image resolution. Volumes from decreasing voxel sizes were increasingly similar to the measured biomass, up to a point: The distribution of voxel sizes of 5 mm more closely matched the biomass distribution than the 2 mm voxel sizes. This may indicate that the relative scale of obscured volume (whether AGB or void) is increased and that smaller voxel sizes may be more representative of the surface of the vegetation. The density of such plant communities may be beyond the limits of the spatial resolution of the imagery (Wallace et al. 2017) or that the differences between shaded and unshaded areas cause issues with the SfM reconstruction process. A more thorough investigation to this spatial grain issue with optical passive imagery, perhaps contrasted with terrestrial or UAS‐based lidar, would further develop insights to the limitations of the volume–biomass relationship of the fine vegetation and litter layers found in this and similar landscapes.

### 4.3 Ground and Vegetation Classification and Separability in Point Clouds

Vegetation in the Three Fingers Allotment can often contain a dense layer of dead medusahead from previous growing seasons, or a thick cover of living medusahead, or both. This means that passive remote sensing techniques such as SfM may have difficulty measuring both internal volumes and the ground surface. Such obstructions likely decreased the correlation between the calculated volume and measured biomass. Iterative classification of the ground surface points increased the observed relationship between volume and biomass for all combinations of vegetation types and biomass. In addition to occluding the ground surface, the layer of litter also confounded the ability to accurately characterize the ground surface. Our classification method extends the classification of point clouds down to the soil level. However, in the case of our reference point clouds (SfM reconstruction after destructive harvesting), traces of litter or vegetation may still be present, as illustrated in Figure S1. It is important to note that rocks are not removed during the classification process but may be eliminated during the destructive harvesting procedure. This introduces some uncertainty for certain point clouds. Furthermore, an additional source of uncertainty in our process stems from the fitted surface on the ground class and the actual ground. Due to gaps in the ground point clouds, caused by occlusions of dense vegetation or litter in the SfM reconstructions, the fitted ground surface may not accurately depict the entirety of the real ground surface as the extrapolation and surface fitting rely on a restricted set of visible ground points within the point cloud. However, the similarity of *R* ^2^ values (0.30 and 0.33, 0.42 and 0.41) and RMSEs (624 and 576 g, 642 and 651 g) between similar model comparisons (all nonground point voxel volumes and convex hull, respectively)—but using reconstructed volumes from all nonground points, and using reconstructed volumes from only those points falling above the reconstructed ground surface—may support our assertion that our ground classification performs well, and few points were removed.

The presence of a dense litter layer on the ground makes it challenging to capture the mineral soil ground surface using passive optical imagery, leading to underestimation in vegetation volume when utilizing voxel volumes: Litter made up an average of 60% of AGB by weight. Figure S2 demonstrates a good fit when comparing the convex hull volume above the reference with the convex hull volume above the reconstructed surface. The convex hull effectively covers volume gaps, accounting for the possible removal of rocks and any remaining litter or vegetation on the reference surface, in close proximity to the two fitted surfaces. However, it fails to address the presence of dense litter within the plot, and we continue to observe underestimation for certain plots.

Our comparison between our reconstructed ground surfaces with the “true” reference ground surface elucidates two points. First, the decrease in predictive ability between the reference surface and our reconstruction is only 2% (from *R* ^2^ of 0.44 to *R* ^2^ of 0.42). This demonstrates that uncertainty in the volumetric calculations using our ground and vegetation classification is constrained. Second, the range of litter biomass in this regression ranged from 20% to 100% of the total vegetation biomass, with evenly distributed errors. While the proportion of litter in the total biomass causes disagreement between the calculated volume in the reference and reconstructed surfaces, the difference does not change the biomass estimated in our model. In sum, this indicates that the disagreement between calculated volumes is independent from the proportion of litter in the total biomass. Thus, it may be possible to adequately quantify uncertainty in volumetric measurements at pasture‐scales. It is possible that the observed relationship is related to the compaction of the litter layer over time as not all volumes are of the same dry weight density. However, a larger and more varied sample size at the smaller plot scale would help further understand this relationship.

### 4.4 Volume Relationships With Vegetation Type Biomass

Because the per‐year and per‐site regressions did not have significant relationships (excepting 2020–2021 South Camp Kettle South, *n* = 36, *R* ^2^ = 0.62, RMSE = 273 g, 5 mm voxel size), we hypothesize that the conditions for all fields were similar and that the reconstructed point clouds were of similar quality and captured the variability of vegetation types equally across the study environment. We observed no significant relationship between vegetation types (e.g., annual and perennial grasses, annual grasses only) and volume calculations. The fidelity of the reconstructed point clouds to the vegetation structure was mostly influenced by whether the ground surface was visible and whether there was a dense layer of litter or stand of vegetation.

Both Cova et al. (2023) and Cooper et al. (2017) had similar difficulty with litter layers in their biomass estimations. Cova et al. (2023) found weak relationships between SfM measurements and biomass in litter‐dominated plots due to occlusion. Cooper et al. (2017) note that neither the SfM nor the TLS could resolve the litter layer. Building on these studies, our iterative technique of identifying ground surface points advances the ability to characterize the ground surface.

### 4.5 Considerations for Future UAS‐Derived and NonDestructive Biomass Data

Our study helps inform UAS‐derived spatial and structural data collection in two ways. Primarily, the unique characteristics of the vegetation and ecological communities in this study area (perennial and annual grass‐dominated, of varying densities and heights, and with significant layers of litter cover) strongly favor higher‐resolution UAS data. While it is not uncommon for UAS sensors to routinely output models at < 5 cm pixel sizes, our analysis underscores the importance of collecting data with pixel sizes closer to 1 cm to ensure precise volumetric estimates of biomass in such environments, as found by Cunliffe, Brazier, and Anderson (2016); Cunliffe et al. (2022). Secondarily, the complications of our analysis, given the common occlusion of the ground surface, highlight that volumetric estimations on such small scales (e.g., 1–10s of cm in litter layers, typically with vegetation heights under 1 m) are sensitive to the spatial resolution of the remote sensing data. While a digital elevation model (DEM) from airborne lidar is typically considered a high resolution for representing the ground surface, its accuracy at scales relevant to this vegetation depends heavily on how it is generated, particularly in areas with dense vegetation where ground points may be obscured.

Given the uncertainty of identifying ground surface, whether from classified points or a reconstructed surface, our proposed method would benefit from refinement before being considered a replacement for destructive harvesting. Such refinements might include systematic comparisons with other commonly used biomass sampling techniques for grasslands such as rising plate meter devices. Separating ground from vegetative biomass, or litter from ground and standing biomass, may benefit greatly from using calibrated multispectral imagery. Uniform and optimal lighting conditions (e.g., diffuse light, similar illumination angles) between all of the sampled plots may enable the RGB values to be used, even if uncalibrated, especially for areas where the dry vegetation and litter are more readily discernible between green vegetation. Active remote sensing techniques such as lidar, whether in conjunction with imaging or alone, may also more reliably return ground points through litter layers and dense vegetation, as well as providing other data such as intensity that may enable greater discernment between ground, litter, and other vegetation.

Other avenues to explore with our proposed method may include additional calculations and measurements from the dense and high‐resolution point clouds, such as vegetation height or other allometry (Cunliffe et al. 2022; Schulze‐Brüninghoff, Wachendorf, and Astor 2021). High spatial resolution of AGB obtained from these point clouds will also enable larger landscape‐scale measurements of AGB, offering a more comprehensive understanding of carbon stocks and ecosystem dynamics. This may include relationships between biomass and structure, or as a more direct replacement of other field‐measured data such as canopy cover. With other passive sensor types such as multispectral or hyperspectral imaging, or active sensors such as lidar, other metrics such as moisture level or classifications may be possible. The fusion of such data types has shown greater success when combined, particularly in grasslands, for predicting not only biomass but also other properties like moisture content (Schulze‐Brüninghoff, Wachendorf, and Astor 2021).

## 5 Conclusions

In this paper, we explored using SfM at ultra‐fine scales in an environment dominated by exotic annual grasses and associated litter. Understanding the amount of biomass and litter in rangeland environments similar to the study site presented here is important for managing forage and fuels and understanding treatments and changes. UAS offers the possibility to scale‐up traditional field methods to larger areas, yet challenges still remain. This research aims to develop field methods and processes to understand the limits of using SfM in semi‐arid ecosystems to estimate biomass. Our research adds to this process by establishing a methodology to further collect and process small plots of biomass into classified point clouds in a largely automated fashion, so that additional work may be conducted and expanded. Our results indicate that the ability to detect the ground may be the limiting factor in attaining satisfactory volume‐to‐biomass relationships in similar environments and reiterating that fine spatial resolutions (mm to cm scale) are needed for fine‐scale vegetation such as *Taeniatherum caput‐medusae* and *Bromus tectorum* .

## Author Contributions

**Josh Enterkine:** conceptualization (equal), data curation (lead), investigation (equal), methodology (equal), resources (equal), software (equal), supervision (equal), validation (supporting), visualization (supporting), writing – original draft (equal), writing – review and editing (lead). **Ahmad Hojatimalekshah:** data curation (supporting), formal analysis (lead), methodology (equal), software (equal), validation (lead), visualization (lead), writing – review and editing (equal). **Monica Vermillion:** investigation (equal), methodology (equal), software (equal), writing – original draft (lead). **Thomas Van Der Weide:** software (equal). **Sergio A. Arispe:** conceptualization (equal), data curation (equal), funding acquisition (lead), investigation (equal), project administration (lead), resources (equal), supervision (equal). **William J. Price:** data curation (equal), investigation (equal), methodology (equal), resources (equal), supervision (equal). **April Hulet:** conceptualization (equal), funding acquisition (supporting), investigation (equal), methodology (equal), resources (equal). **Nancy F. Glenn:** conceptualization (equal), funding acquisition (equal), methodology (equal), project administration (equal), supervision (equal), writing – review and editing (equal).

## Conflicts of Interest

The authors declare no conflicts of interest.

### Open Research Badges

This article has earned an Open Data badge for making publicly available the digitally‐shareable data necessary to reproduce the reported results. The data is available at https://doi.org/10.18122/bcal_data.8.boisestate.

## Supporting information


Data S1.

## Data Availability

A portion of the imagery (35 plots with both pre‐ and post‐destructive harvesting data), our Python script for SfM batch processing containing processing parameters, generated point clouds used in our analysis, biomass data, and the Matlab script for ground classification are available via Boise State University Scholarworks, DOI: https://doi.org/10.18122/bcal_data.8.boisestate
