# Supplementary material for: Voxel Volumes and Biomass: Estimating Vegetation Volume and Litter Accumulation of Exotic Annual Grasses Using Automated Ultra‐High‐Resolution SfM and Advanced Classification Techniques
Source: Ecol Evol. 2025 Jan 23;15(1):e70883. doi: 10.1002/ece3.70883 (PMC11756975; doi:10.1002/ece3.70883)
Supplement: Supplementary file 1 — Data S1. [file ECE3-15-e70883-s001.docx]

Supplementary Table 1. All Volume and Biomass Relationships.

| Relationship | Linear regression R^2^ | Log regression R^2^ |
| --- | --- | --- |
| Vegetation voxel volume vs AG biomass | 0.274 | 0.287 |
| Vegetation Convex hull volume vs AG biomass | 0.124 | 0.228 |
| Regression between AG biomass percentage from the field and vegetation convex hull volume | 0 | 0.02 |
| Regression between PG biomass percentage from the field and vegetation convex hull volume | 0 | 0.02 |
| Regression between AG biomass percentage from the field and vegetation voxel volume | 0.01 | 0.02 |
| Regression between PG biomass percentage from the field and vegetation voxel volume | 0 | 0 |
| Regression between AG+PG biomass percentage from the field and vegetation voxel volume | 0 | 0 |
| Vegetation voxel volume vs AG+PG biomass | 0.327 | 0.399 |
| Plot-level voxel volume vs AG+PG biomass | 0.321 | 0.387 |


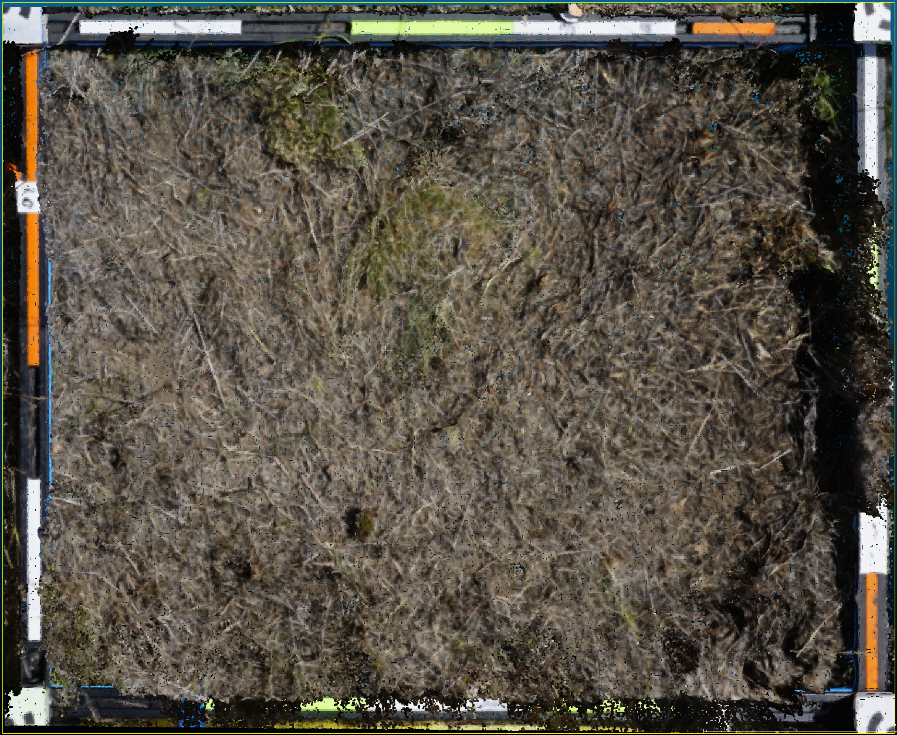


Supplementary Figure 1. Point cloud of the ground following vegetation removal.


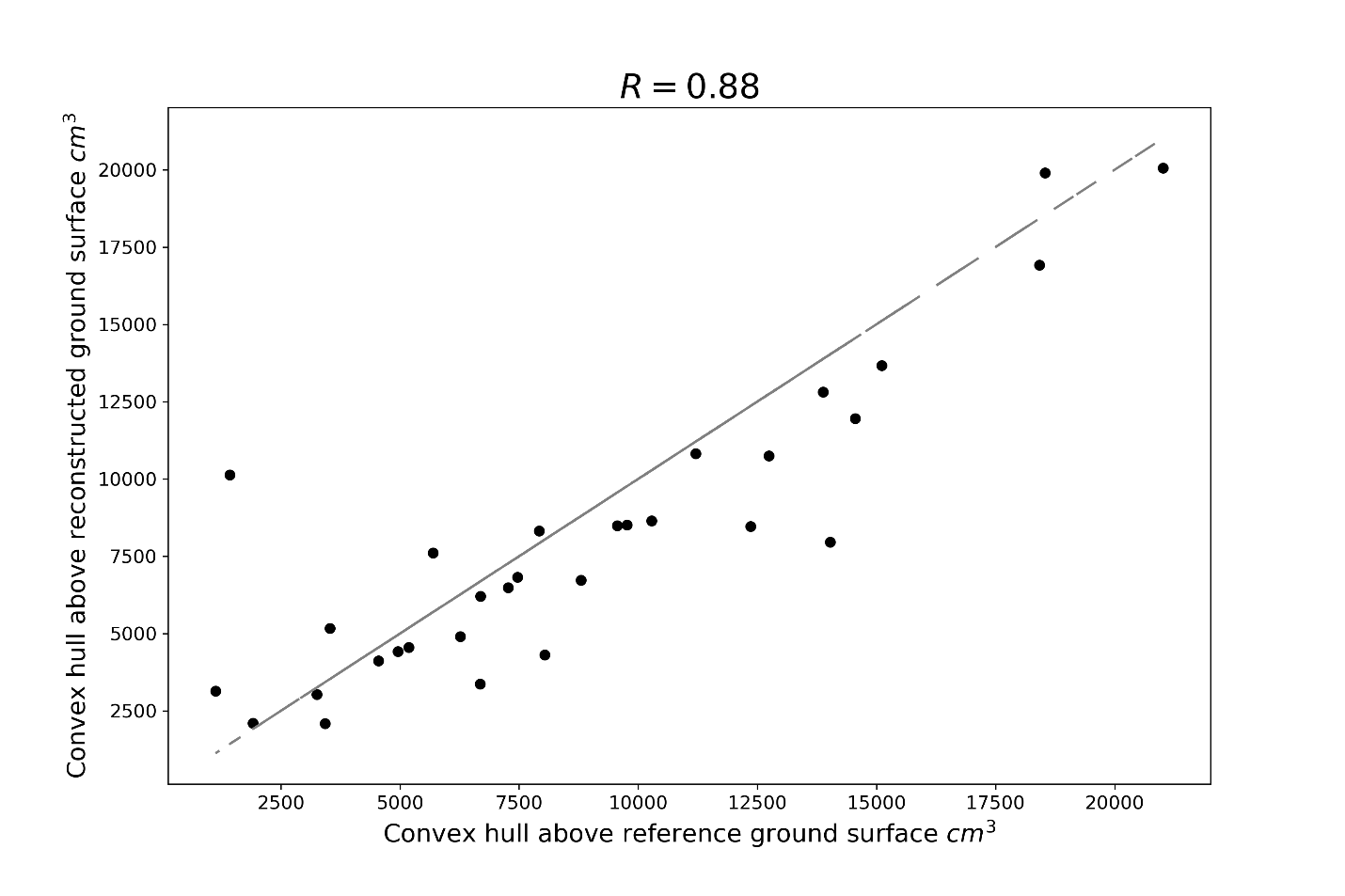


Supplementary Figure 2. Calculated vegetation and litter convex hull volumes using reference ground surface and reconstructed ground surface.
